# A Case Presentation of Three Polypoid Melanomas With Divergent Features

**DOI:** 10.7759/cureus.46951

**Published:** 2023-10-13

**Authors:** Mikalah E Maury, Drazen M Jukic

**Affiliations:** 1 Dermatopathology, Mercer University School of Medicine, Savannah, USA; 2 Dermatopathology, Georgia Dermatopathology, Savannah, USA

**Keywords:** dermatopathology, dermatology case report, clinical dermatology, melanoma with divergent features, metastatic melanoma, polypoid melanoma, rare melanoma, nodular melanoma

## Abstract

Polypoid melanoma, a subtype of nodular melanoma, is classified as the most aggressive and deadly form of cutaneous melanoma. Its rapid vertical growth phase and a wide array of divergent features make clinical diagnosis extremely difficult. This report includes three cases of polypoid melanoma that were all originally thought to be other benign lesions or non-melanoma cancer. These cases feature the variability of the clinical presentation of polypoid melanomas while emphasizing the importance of an annual skin examination, complete lesion biopsies, and working with experienced dermatopathologists for the correct diagnosis and prompt treatment of these cancers. By sharing these cases and general information on polypoid melanoma, we aim to spread awareness of this rarer subtype of melanoma and highlight the importance of having a broad differential list when presented with suspicious lesions.

## Introduction

Nodular melanoma is the second most common subtype of malignant melanoma after superficial spreading and makes up approximately 15%-20% of primary cutaneous melanoma diagnoses [1,2]. A subtype of nodular melanoma, the polypoid type, is understood to have an almost nonexistent radial growth phase followed by an aggressive and rapid vertical growth phase in which at least half of the lesion is located above the epidermis [3]. This presentation can cause the malignancy to be mistaken for other lesions such as pyogenic granuloma, fibroepithelial polyp, hemangioma, intradermal nevus, cutaneous metastases, or even an infectious process [4]. Histologically, high levels of cellular and nuclear pleomorphism, cellular atypia, and a high mitotic index account for its poor prognosis. In addition, early metastases via vasculature and lymphatic invasion make polypoid malignant melanoma the deadliest of all melanomas [4,5]. Here, we report three cases of polypoid melanoma with divergent features that demonstrate its difficulty to diagnose clinically and the possible ways to avoid misdiagnosis.

## Case presentation

Case report 1

**Figure 1:**
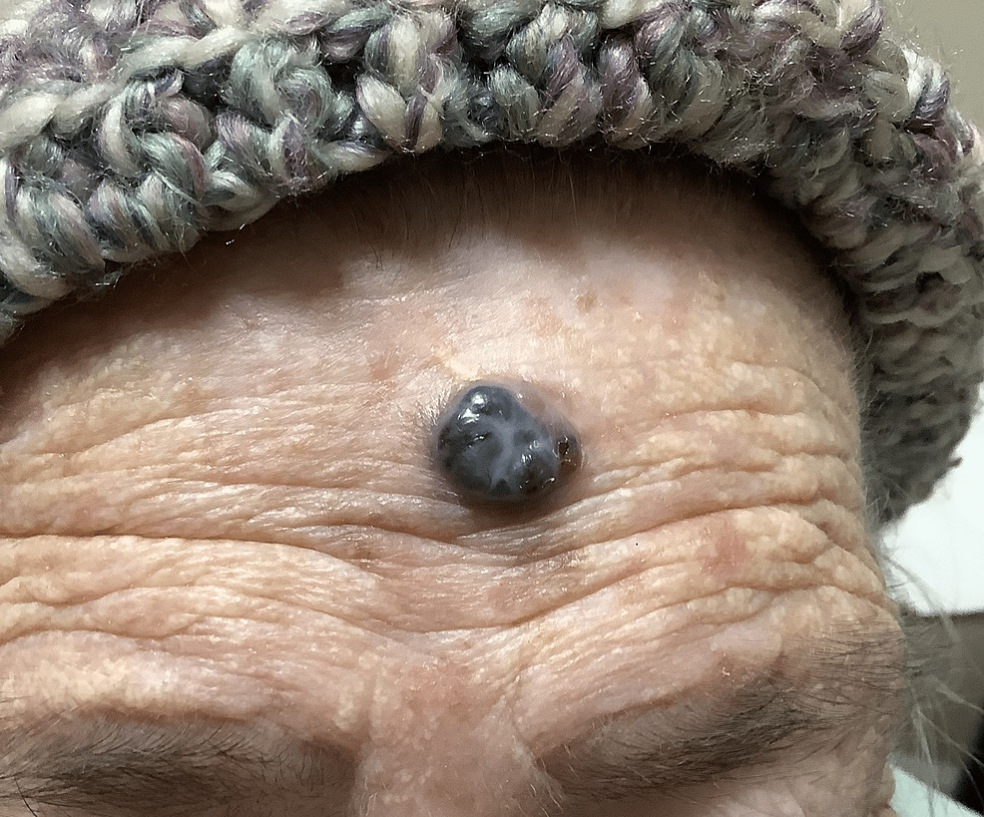
Image of the patient from case report 1 showing a large, dark polypoid melanoma of the forehead that was previously obstructed by the knit hat.

**Figure 2:**
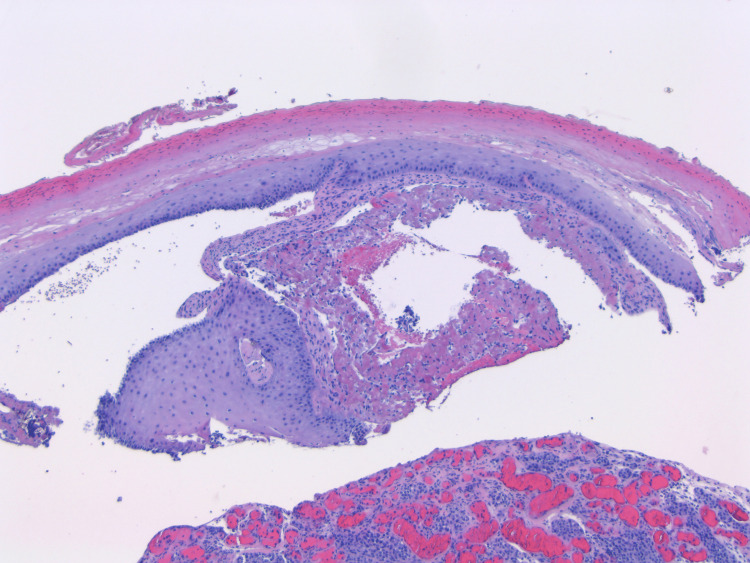
2× H&E: There is a complete dermoepidermal separation with serum deposit between the dermis and epidermis. There is no melanoma seen in the epidermis at hand. H&E: hematoxylin and eosin

**Figure 3:**
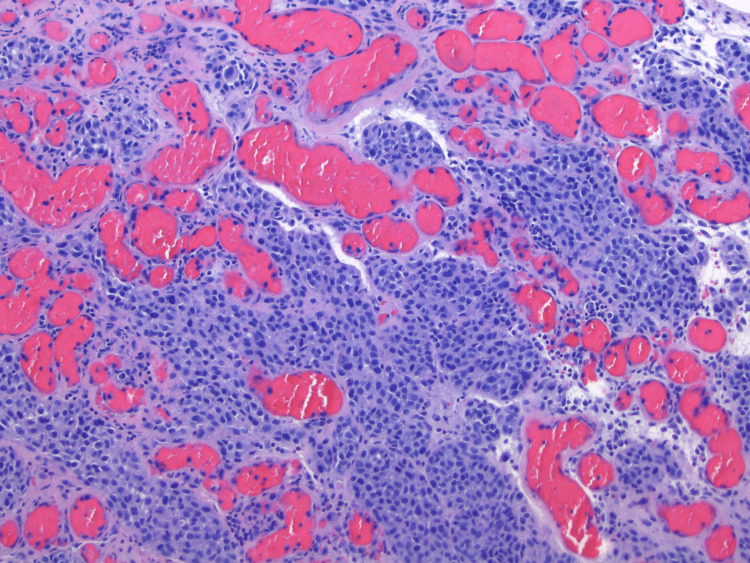
10× H&E: Higher power view of Figure 2. Malignant small melanocytes, partially nested, are seen in the immediate vicinity of the dilated vasculature. There is no clear-cut involvement of the vessels seen by the neoplasm. H&E: hematoxylin and eosin

**Figure 4:**
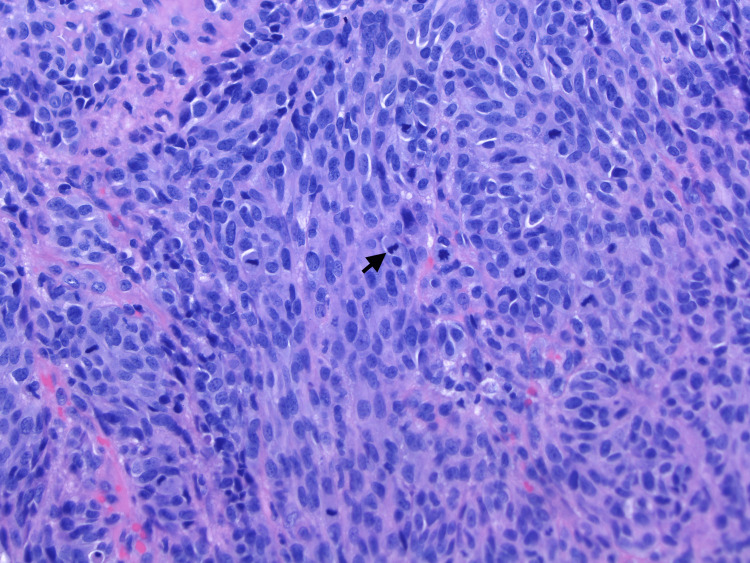
20× H&E: Higher power view of Figure 3. Numerous mitotic figures are seen throughout the neoplasm. The arrow points to the mitotic figure. H&E: hematoxylin and eosin

An 88-year-old female with a history of diabetes and smoking originally presented with a large erythematous, ulcerated, nodular growth of the left nasal ala that compressed the nostril’s airway and had been growing for several years. The patient had no previous non-melanoma or melanoma skin cancer history. Through examination, it was determined that a biopsy was necessary and, after pathology review, was determined to be a well-differentiated basal cell carcinoma. Upon further examination and removal of a knit hat the patient was wearing, a second large purplish-black nodular lesion was identified on the medial forehead (Figure 1). The patient reported the lesion had also been there for many years but recently had changed in size by growing further above the skin and color over time. Initial differentials included a blue nevus, hemorrhagic bulla and pyogenic granuloma due to its substantial vertical growth and, at least clinically, fluid-filled appearance. A closer look revealed a firm, multicolored lesion. With a new suspicion for a malignant melanoma, an excisional biopsy was done. The pathology report showed a polypoid malignant melanoma, blue nevus-like, with a Breslow thickness of 5.5 mm and Clark level V (Figures 2-4). Additional prognostic classification via DecisionDx-Melanoma molecular testing placed the lesion in class 1B, which is associated with an increased risk of recurrence/metastasis within five years. One month after her office visit, the patient had a stroke and was hospitalized for an unknown length of time. She was able to recover and was discharged to a nursing home where she still currently resides. Due to her age and current quality of life, she reported that she was not interested in having any surgeries or further treatment at this time, including a sentinel lymph node biopsy.

Case report 2 

**Figure 5:**
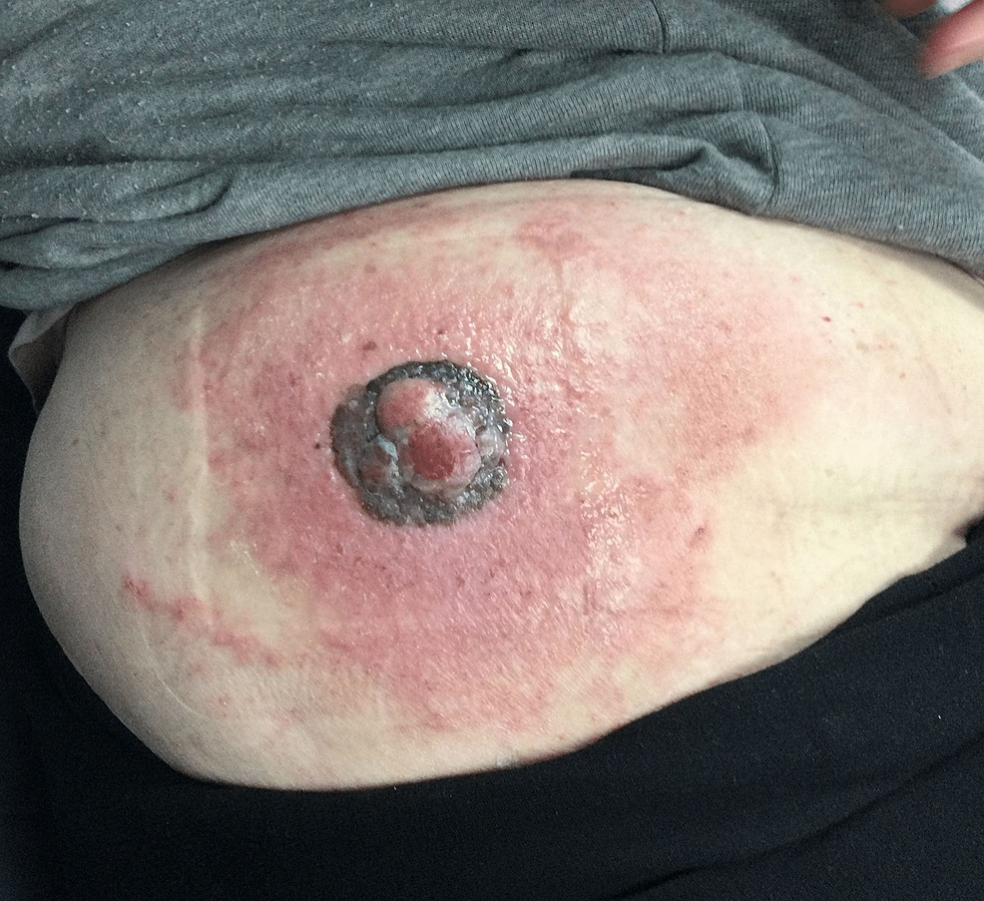
Central polypoid lesion surrounded by a large, black-gray central plaque flanked by erythema and scaling.

**Figure 6:**
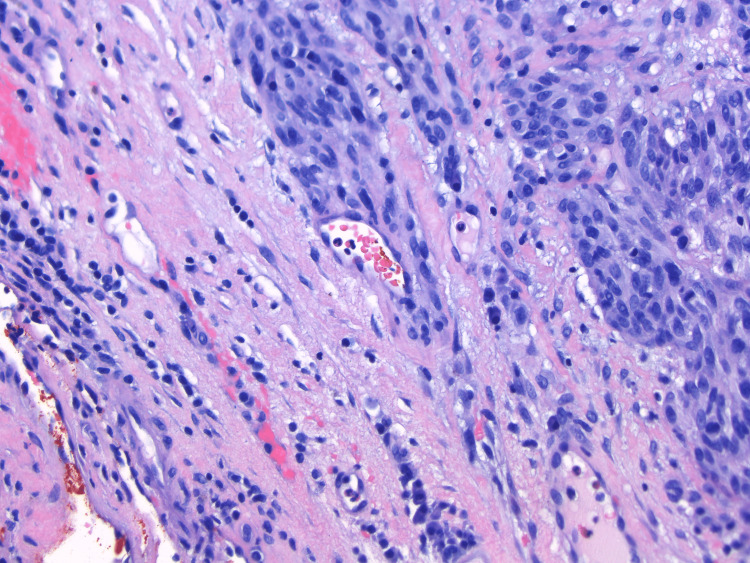
20× H&E: Melanoma is seen extending in a perivascular fashion in the deep dermis. H&E: hematoxylin and eosin

**Figure 7:**
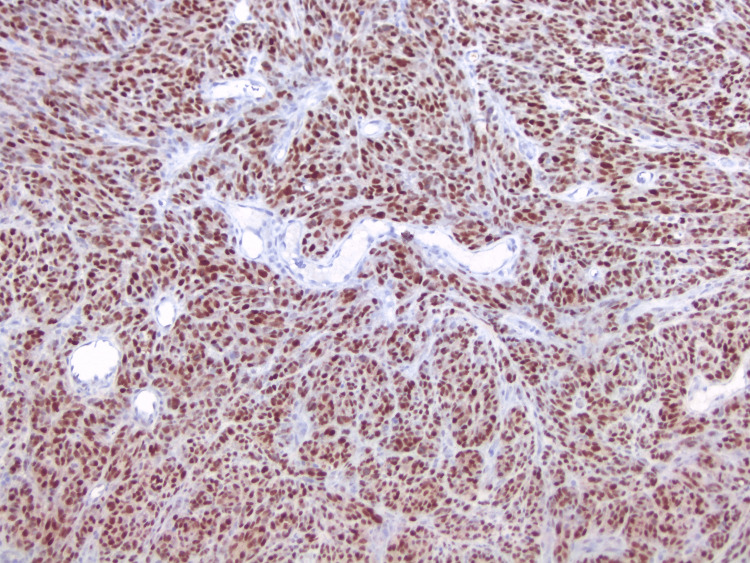
Reactivity for PRAME-HMB-45 antibody (10×): All the brown nuclei are PRAME-positive, which is a tumor-associated antigen expressed by malignant melanocytic lesions. HMB-45 (which is cytoplasmic and would be red in this preparation) is completely negative. PRAME: PReferentially expressed Antigen in MElanoma

**Figure 8:**
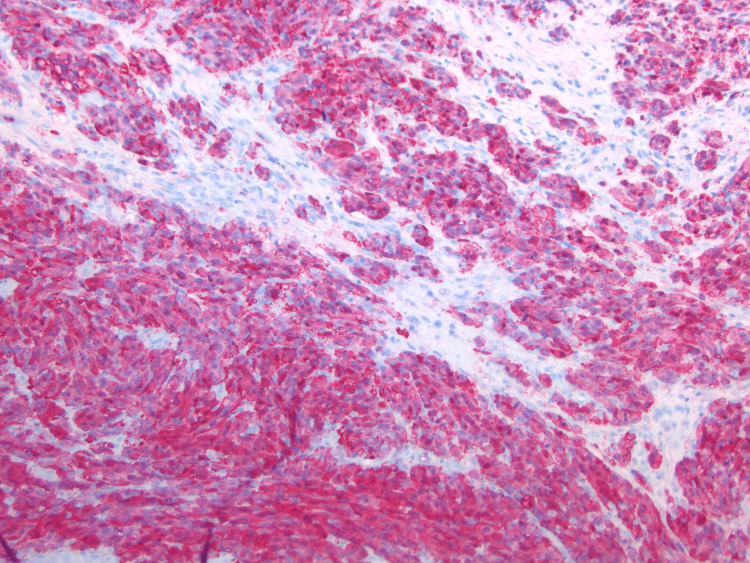
Reactivity for S100 (10×) (the most sensitive marker for melanocyte differentiation): Intense positivity with both cytoplasmic and nuclear reactivity.

A 47-year-old female with a history of hypertension presented with a large, black/gray ulcerated plaque with an erythematous raised central nodule and surrounding erythema with scaling approximately 5-6 inches at its greatest dimension, around the right flank (Figure 5). The patient had no prior history of non-melanoma or melanoma skin cancers and no pertinent family history. The patient stated the lesion “started as a raised mole” and then increased in vertical size before spreading outward over the past two years. The patient also stated that about two months ago, she injured the area while at work, and it “got infected.” Over the last two weeks before the office visit, it progressively became more painful and bled. The primary differential was cellulitis with necrosis and inflammation secondary to trauma. Clear cell sarcoma of soft parts and malignant peripheral sheath tumor were also considered being less likely differentials. A 4 mm punch biopsy was done, the lesion was packed with gel foam, and a pressure bandage was applied. A prescription for minocycline 100 mg was given for possible infection, and triamcinolone 0.1% was prescribed for the scaling process. The pathology report revealed a polypoid malignant melanoma, markedly ulcerated, with a Breslow thickness of at least 1.1 cm and Clark level of at least IV (deep tissue edge was broadly involved) (Figures 6-8). Further prognostic classification via DecisionDx-Melanoma showed a 2B class score, which has the highest risk of recurrence/metastasis within five years. The patient was immediately referred to surgical oncology for further treatment. After the initial consultation with oncology, she was lost to follow-up. A sentinel lymph node biopsy was scheduled, but the patient did not return for treatment, and no further therapy is known. We attempted to contact her for further history to no avail.

Case report 3

The patient is a 92-year-old male with a history of hypertension, diabetes, stroke, anemia, thrombocytopenia, and chronic ischemic heart disease. Over the span of 12 years, the patient had a skin-relevant history of squamous cell carcinoma (SCC) (left lower extremity), two melanomas in situ (right abdomen and left lower back), and an invasive malignant melanoma (right upper back, Breslow thickness: 0.8 mm, Clark level: IV). The last two biopsies performed on him revealed keratoses, and he had not returned to the dermatologist for two years.

**Figure 9:**
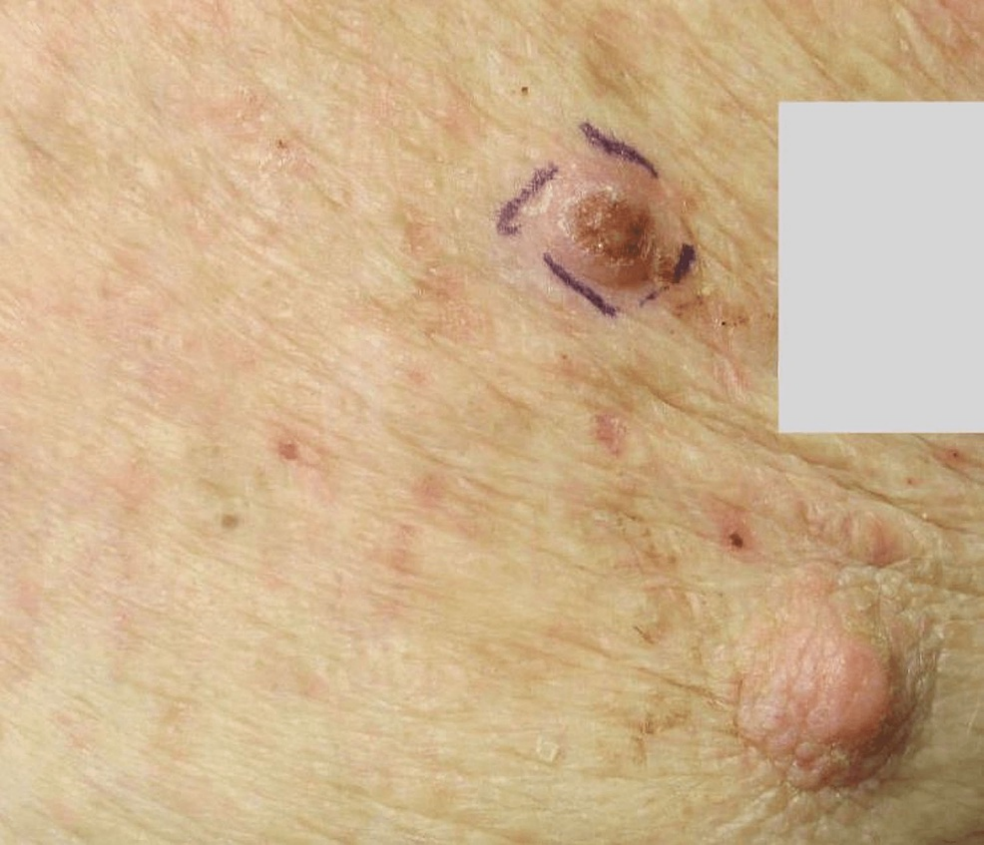
Image from case report 3 of the polypoid melanoma: The left nipple in the bottom right corner was included for orientation and size reference.

**Figure 10:**
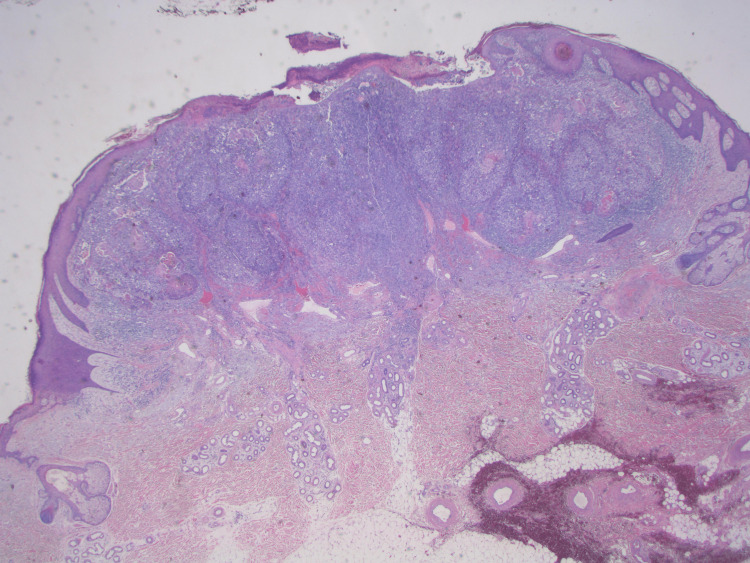
1.25× H&E: Low power view of the entire lesion. There is prominent growth of squamous epithelium seen with central nodular growth of clear cells. H&E: hematoxylin and eosin

**Figure 11:**
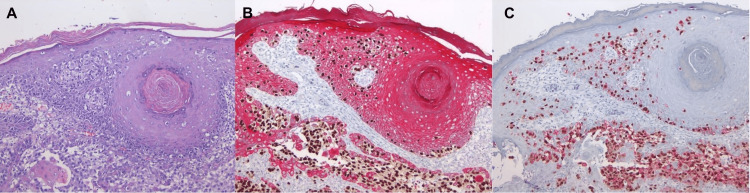
Composite image of three different antibodies (H&E (A), Sox10/AE1A3 (B), and PRAME-HMB-45 (C) all at 4×): This area, which is in the epidermis, reveals areas of in situ melanoma, reactive with Sox10, partially reactive with HMB-45, and strongly reactive with PRAME while being nonreactive with A1AE3. In the dermis underneath the epidermis, there is a larger deposit of invasive melanoma, still arising and accompanied by prominent epidermal hyperplasia. PRAME: PReferentially expressed Antigen in MElanoma

In 2018, he presented with a nodular lesion on the left chest, which was ulcerated and easily bled; the surrounding skin was red and swollen (Figure 9). This nodular neoplasm was removed by excisional biopsy and originally interpreted as “squamous cell carcinoma, margins free of tumor.” However, during the random case review in 2020, the original diagnosis of squamous cell carcinoma was questioned, and an additional opinion by a dermatopathology expert was sought. After performing appropriate immunohistochemical antibodies, a modified report was issued, revealing an amelanotic, polypoid melanoma that was mostly in situ, along with an area of invasion. There was, however, florid reactive squamous hyperplasia, inverted follicular keratosis-like, thus elevating the Breslow thickness to 2.9 mm and Clark level to V (the only invasive area was at the bottom edge, and melanoma invaded the subcutaneous fat) (Figures 10, 11). We attempted to contact the patient to check on the status of the lesion but were informed by the patient’s son that he had passed away six months after the visit in 2018 from cardiac complications.

## Discussion

We present three different cases of polypoid, nodular melanoma, all of which had unique and divergent features from one another and clinicopathologic features not usually associated with the initial diagnosis of melanoma in modern-day patients. Nodular melanomas often fail to fulfill the diagnostic ABCDE (asymmetry, border, color, diameter, and evolving) rule for clinical recognition by being more symmetrical in early stages, having a smaller radial size, and presenting with only one color. Polypoid type, in terms of both gross anatomy and histology, reflects a tumor where more than half of the tumor exists largely above the skin surface [5].

In case 1, there was symmetrical and significant vertical growth more typical for a polypoid melanoma. There was also a homogeneous navy color throughout the lesion, however, initially indicating a benign process on clinical diagnosis. In contrast, case 2 started as a small raised “mole” initially before a more aggressive radial spreading lesion developed with multiple colors throughout, as well as a substantial ulcer. It is our hypothesis that the polypoid cancer began as a lesion primarily above the skin surface; however, due to the two-year delay in the patient seeking care for the lesion, it progressed to a melanoma that was unrecognizable as polypoid type. Even more interesting was the large area of surrounding erythema, which, considering the severity and thickness of this malignancy, indicates in-transit metastases as the melanoma spreads out via proximal vasculature and lymphatics [1]. This was further confirmed upon review of the pathology report indicating perivascular infiltration (Figure 6). Frequently, nodular melanomas can be amelanotic when compared to more superficially spreading lesions [6]. In fact, one study reviewing the incidence of nodular melanomas found that in 60% of cases reviewed, there was no pigmentation evident to cause concern for a melanoma, such as described in case 3 [2]. The unique aspect of this case, with the additional area of squamous hyperplasia within the lesion, further leads to the initial misdiagnosis and a subsequent increase in both Breslow thickness and Clark level. It is also important to note that while polypoid melanomas can be pedunculate and cauliflower-shaped with a stalk, this is not the case for all polypoid lesions. Polypoid, in essence, means dome-shaped and elevated, with most of the cancer above the level of the epidermis due to its vertical growth. Therefore, it is necessary to continue to evaluate the patient’s history regarding the lesion to understand how it initially presented and how it has progressed. By obtaining the information that case 2 and 3’s lesions started as singular raised lesions, the polypoid subtype was able to be considered higher on the differential.

## Conclusions

Avoiding partial biopsies of lesions and working with an experienced dermatopathologist are crucial for the correct diagnosis and prompt treatment of polypoid, nodular melanomas. Melanoma makes up only 1% of skin cancers diagnosed but is the leading cause of skin cancer mortality in the United States. Because of the ability of nodular melanomas to resemble many different benign lesions and even non-melanoma skin cancers, it is necessary for clinicians to expand their list of differentials, advocate for annual skin examinations in high-risk patient populations, and utilize tools such as dermoscopy when evaluating lesions. Specifically, polypoid melanomas on visualization with dermoscopy typically present with a multicolored pattern, polymorphic vessels, and the so-called “fiber sign,” where these raised lesions snag on clothing or hair and obtain those fibers attached to the lesion. Lastly, it is paramount that clinicians adapt their threshold for biopsy with any suspicion of a possible melanoma, especially early nodular types.
